# Adolescent nicotine exposure and persistent neurocircuitry changes: unveiling lifelong psychiatric risks

**DOI:** 10.1038/s41380-025-03110-0

**Published:** 2025-08-29

**Authors:** Lauren M. Reynolds, Philippe Faure, Jacques Barik

**Affiliations:** 1 Plasticité du Cerveau CNRS UMR8249, École supérieure de physique et de chimie industrielles de la Ville de Paris (ESPCI Paris), Paris, France; 2 Neuroscience Paris Seine CNRS UMR 8246 INSERM U1130, Institut de Biologie Paris Seine, Sorbonne Université, Paris, France; 3 CNRS – UMR 7275, Institut de Pharmacologie Moléculaire et Cellulaire, Valbonne, France; 4 Université Côte d’Azur, Nice, France; 5 Inserm U1323, Valbonne, France

**Keywords:** Neuroscience, Psychiatric disorders

## Abstract

Nicotine exposure during adolescence has emerged as a significant risk factor for later psychiatric disease. Notably, adolescence is a critical period for the maturation of acetylcholine and dopamine systems, neuromodulators which tightly regulate cognitive, motivational and emotional behaviors known to contribute to psychiatric vulnerability. This review explores whether long-lasting modifications in these neuromodulatory systems following adolescent nicotine exposure underlie the increased vulnerability to mental health disorders. We discuss evidence that nicotine in adolescence leads to enduring molecular, cellular alterations by perturbing the normal trajectory of cholinergic and dopamine systems, and link these changes with potential adverse behavioral outcomes in adulthood. We propose that persistent alterations in acetylcholine and dopamine signaling caused by adolescent nicotine exposure may contribute to the heightened risk for psychiatric disorders including substance abuse, anxio-depressive disorders, and schizophrenia for which deficits in a large spectrum of motivational domains are highly prevalent. The interaction between nicotine and these developing neurotransmitter systems during adolescence raises important questions about the mechanisms driving these changes. Finally, we discuss limitations in the current research and subsequently identify open questions in the field which will help drive forward research on the psychiatric consequences of adolescent nicotine use. Understanding these maladaptations could pave the way for targeted therapeutic strategies to mitigate the adverse effects of adolescent nicotine exposure on brain development and subsequent psychiatric outcomes.

## Introduction

Smoking is a major contributor to disease burden worldwide, driven by addiction to nicotine, the primary psychoactive and addictive component of tobacco [1]. Nicotine addiction is a chronic relapsing disorder, and the prognosis may be particularly bleak for the up to 90% of adult smokers who began in adolescence [2], as early onset nicotine use is associated with longer and heavier smoking careers [3], and smokers with an adolescent onset are less likely to quit smoking than those who began as adults [4].

In addition, nicotine use is associated with multiple psychiatric comorbidities [5]. While deciphering the nature and direction of the relationship between nicotine use and comorbid psychiatric disorders can be challenging, studies increasingly point adolescent onset nicotine use as a strong predictor for later onset of psychiatric disease. Nicotine use in adolescence has been shown to predict the later appearance of depression symptoms [6–10]. Cigarette smoking in adolescence has also been associated with the onset of various anxiety disorders, which include generalized anxiety disorder, panic disorder, and post-traumatic stress disorder, in early adulthood, even after controlling for the presence of anxiety and depressive disorders in adolescence [11], and is also associated with a shorter time to onset of anxiety disorders [12]. Nicotine is also posited to act as a “gateway drug”, where its use in adolescence is linked with later abuse of different drug categories, such as alcohol, opiate drugs, or cocaine [3, 13–15]. These mental health disorders are a leading contributor to global disease burden, substantially reducing quality of life [16, 17].

Nicotine in adolescence may produce enduring psychiatric vulnerability by interfering with ongoing neurodevelopmental processes. Indeed, adolescence is a period of dramatic brain maturation when the continued formation and pruning of synaptic connections refines precise neuronal networks. Neuroimaging approaches have begun to shed light on complex maturational processes in both healthy subjects and pathological cases. Indeed, evidence from cross sectional and longitudinal structural MRI studies of human brain development show that gray matter thickness in cortical regions decreases across adolescence before stabilizing at adult levels, while white matter volumes increase [18–21]. Postmortem studies suggest that these macroscale changes likely reflect cellular, molecular, and connectivity development [22–25]. Functional imaging studies further indicate that significant changes occur in the function of subcortical structures and in their relationship with cortical regions, notably in nuclei associated with reward and motivation [26–29]. These neurobiological changes underlie the profound maturation in motivational and cognitive domains across adolescence [30–34], and interfering with these processes may bias individuals toward a vulnerable psychiatric state. All of these developmental processes are proposed to be highly sensitive to disruption by environmental stimuli.

In the current review we discuss the progress in our understanding of how nicotine in adolescence increases later addiction and psychiatric vulnerability, with a focus on recent advances in understanding how nicotine in adolescence enduringly alters the molecular, neurophysiological, and properties of discrete neuromodulatory circuits in animal models. Given the predominant role that dysregulations in motivational and cognitive processes play in psychiatric conditions, we place a specific emphasis on enduring neuroadaptations in cholinergic and dopaminergic circuits following adolescent nicotine exposure.

## Adolescent development of neuromodulatory systems in translational models

**Fig. 1:**
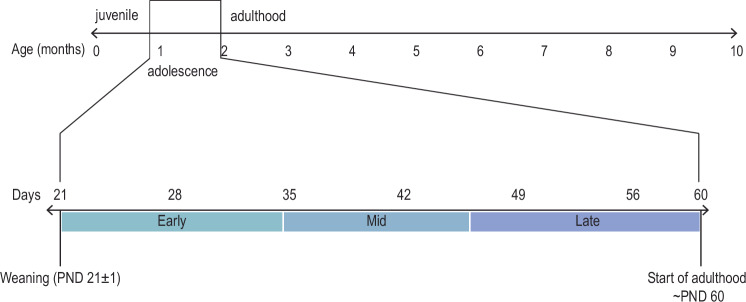
Situating adolescence in the rodent lifespan. Adolescence can be considered to last from weaning (generally PND 21) until rodents achieve complete sexual, behavioral, and neurobiological maturity around PND 60. It can be further divided into early (~PND 21–34), middle (~PND 35–45), and late (~PND 45–60) periods where animals exhibit different epochs of sexual development and exhibit certain behavioral and neurobiological characteristics.

While this period is most often thought of in the context of human teenagers, it is increasingly recognized to be evolutionarily conserved, with animal species also showing distinct markers of an adolescent state [35–40]. Defining the boundaries of adolescence has proved challenging, as there are not clear hallmarks to signal the onset or completion of adolescent development. While puberty and adolescence occur during the same time period, they are not necessarily synonymous: puberty represents the process of attaining sexual maturity, while adolescence is a more diffuse period marked by significant neurobiological, behavioral and social changes. Thus, the age boundaries of the adolescent period are difficult to precisely define. Modern definitions of the human adolescent period range between 10 and 24 years of age [35, 41], an age range where children show dramatic behavioral changes and the human brain undergoes significant restructuring [22, 23, 42, 43]. While there is no clear consensus on the age range of adolescence in animal models, adolescence in rodents is increasingly defined more in line with this larger age window in humans, by counting the period from weaning (~PND 21) where mice and rats gain begin to interact independently with their environments for the first time, leading to a wealth of experiences with the potential to enduringly impact adult behavioral phenotypes and their underlying neuronal circuits. Rodents show behavioral and neurobiological markers of development until neurobiological maturity is reached around two months of age (~PND 60) [36, 38, 39, 44–47]. The adolescent period can be further sub-divided into early (~PND 21–34), middle (~PND 35–45), and late (~PND 45–60) periods [39, 46], which coincide roughly with pre- peri- and post-pubertal periods (Fig. 1). It is important to define these time windows, as striking, age-dependent differences can be reported in the enduring effects of the same experience on neural circuitry depending on the adolescent period in which this experience occurred [48–51]. Thus, we take great care in this review to be as specific as possible about the age at which nicotine exposure occurred during adolescence.

**Fig. 2:**
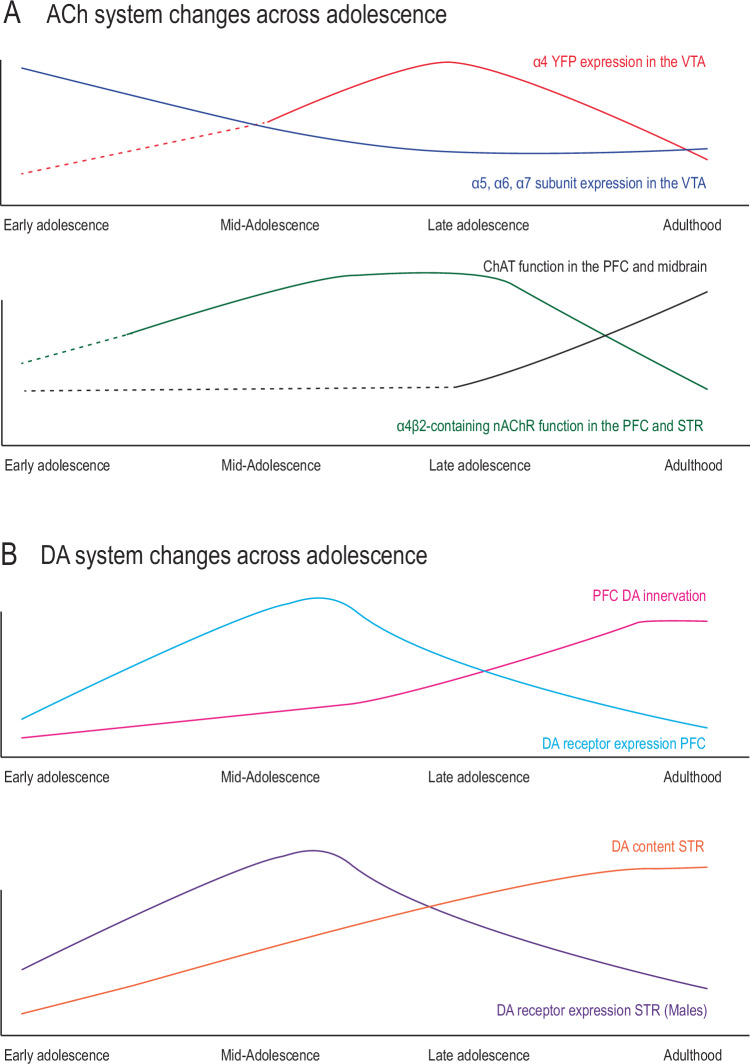
Adolescent changes in acetylcholine and dopaminergic systems. **A** ACh system changes across adolescence. *Top*: regional changes in nAChR subunit expression have been noted across adolescence, with a peak in late adolescence of α4 receptor expression in the VTA, as assessed with YFP tagging, and with a gradual lessening of α5, α6, and α7 receptors in the VTA between adolescence and adulthood. Dotted lines represent periods where expression has not yet been studied. *Bottom*: α4β2-containing nAChR function in the PFC and striatum increases from late adolescence to adulthood. ChAT function changes across adolescence in the PFC and in the midbrain, with a peak in function in mid- to late adolescence before declining to adult levels. **B** Dopamine system changes across adolescence. *Top*: In the PFC, DA innervation increases steadily across adolescence until reaching a plateau around PND 60. DA receptor expression in the PFC peaks in mid-adolescence before declining to adult levels. *Bottom*: DA content in the striatum increases across adolescence, while DA receptor expression peaks in mid-adolescence. This peak appears to be sex specific, as it has been confirmed in male rodents, but was not seen in females.

Studies in rodent models have begun to elucidate the complex development of neuromodulatory neurotransmitter circuits in adolescence, including those most likely to be directly affected by adolescent nicotine use. Nicotine usurps the action of endogenous acetylcholine (ACh) by signaling through nicotinic acetylcholine receptors (nAChRs), a diverse family of pentameric ligand-gated cation channels [52]. While nAChRs are present throughout the brain at all developmental stages, maturational patterns in their expression and function have been identified [53, 54]. Notably, expression of the most abundant and high-affinity α4β2-containing nAChRs across the brain peaks in adolescence before diminishing to adult levels [55–58], and this peak has been suggested to underlie elevated nicotine self-administration in adolescent animals [59]. This increased nAChR expression was notably observed within the striatum and the midbrain [59]. Studies with greater regional specificity have shown that expression of α4- containing nAChRs in the VTA peaks late in adolescence, and is positively correlated with nicotine intake in an oral self-administration task (Fig. 2A) [60]. Rubidium efflux assays, an index of potassium channel activity, indicate higher nAChR receptor functionality in adolescents than in adults in the cortex, striatum, hippocampus, and thalamus (Fig. 2A) [61, 62]. In parallel to the developmental plasticity of cholinergic receptors, activity of choline acetyltransferase (ChAT), a requisite enzyme for ACh synthesis that also reflects the density of cholinergic innervation, increases from adolescence to adulthood in both the cortex and midbrain (Fig. 2A) [63]. At the cellular level, electrophysiological response to nicotine in cholinergic cells from the laterodorsal tegmentum (LDT), a small brain region located in the brainstem that constitutes the primary source of ACh to the ventral tegmental area (VTA) [64, 65], changes across development, with a larger excitatory response in cells from juvenile mice (PND 7–15) than in late juvenile/early adolescent mice (PND 15–34). This change in nicotine responsivity in LDT neurons is thought to represent a developmental reorganization of the nAChR subunits present on these cells [66, 67].

ACh provides a complex regulation of reward and motivation behaviors through its actions on dopamine (DA) circuitry [68–72]. The release of ACh from LDT projections directly modulates activity of VTA DA neurons, finely tuning their firing rate and burst properties [73, 74]. DA signaling in the striatum is also modulated at the terminal level by ACh release from local striatal cholinergic interneurons [75], but also from an external direct cholinergic input from the LDT [76]. Hence, ACh signaling through presynaptic and somato-dendritic nAChRs tightly regulates DA circuit activity, suggesting that it is another neuromodulatory system profoundly affected by developmental exposure to nicotine. Dopamine circuits undergo a robust period of maturation in adolescence, including changes to innervation patterns, dopamine synthesis and release, basal dopamine neuron firing rates, and dopamine receptor expression in terminal areas, with particularly pronounced changes in mesocortical and mesolimbic circuits (Fig. 2B) [39, 77–82]. Of note, the segregation of mesocorticolimbic dopamine pathways continues in adolescence, with a subset of dopamine axons passing through limbic regions before growing to the PFC [83]. The targeting processes of these axons can be disrupted by experience with stimulant drugs of abuse in adolescence, such as amphetamine [49, 51], or by stress [84], resulting in enduring cognitive deficits.

The development of dopaminergic and cholinergic systems may also be inter-related. Adolescent rodents show greater DA release in the NAc in response to acute nicotine when compared to adult counterparts, despite similar baseline measures [85, 86]. Electrophysiological response of VTA DA neurons to acute nicotine also changes across development, with an increased sensitivity in early adolescent animals [87]. Molecular evidence links these changes to maturation in VTA nAChR expression, as mRNA transcripts of α5 α6 and α7 nAChR receptor subunits in the VTA peak early in adolescence (Fig. 2A), in tandem to nicotine-stimulated DA release in the ventral striatum [88]. Together, these results suggest that nicotine in adolescence produces different immediate and long-term outcomes through its actions on these developing neurotransmitter systems.

## Behavioral outcomes of adolescent nicotine exposure in animal models : translational evidence for psychiatric vulnerability

These developmental differences in cholinergic and dopaminergic function may underlie the age-specific behavioral responses provoked by an acute exposure to nicotine. In particular, many studies have shown that adolescent rodents show an increased sensitivity to the locomotor activating and rewarding effects of nicotine, blunted withdrawal symptoms, and reduced aversion to high doses of nicotine, compared to adult counterparts. Furthermore, these behavioral outcomes have been linked to differences in response to nicotine across several neurotransmitter systems, as well as recruiting microglial pathways to sculpt circuitry in response to acute exposure [44, 89–91]. These studies on the age-dependent acute effects of nicotine are outside the scope of the current review, but have been well reviewed in the above citations. How nicotine exposure in adolescence affects later sensitivity to nicotine has also been heavily studied, particularly from a behavioral point of view. Nicotine in adolescence has been shown to increase later sensitivity to the rewarding effects of nicotine, while decreasing its aversive effects; these studies have been thoroughly reviewed elsewhere, see: [90, 92]). Nicotine in adolescence has also been proposed to increase sensitivity to other psychostimulant drugs in adulthood [93], leading, notably, to its reputation as a “gateway drug”.

A second focus of behavioral studies with nicotine in adolescence has been its long-term effects of translational behavioral markers for vulnerability to psychiatric disease [94, 95]. Exposure to nicotine in mid-adolescence (PND 35–44) induces anxiety-like behaviors, such as a reduction of time spent in the open arms of the elevated plus maze, in adult rats and promotes a persistent depressive-like state, with an increased immobility in the forced swim test, and reduced sucrose preference [96, 97]. Notably, the consequences of adolescent exposure to this nicotine regimen were sex-specific, with nicotine-induced vulnerability to anxiety- or depression-like behavior specific to male rats [98]. Anxiety- and depression-like behaviors induced by adolescent nicotine exposure can be rescued in adult male rats by re-exposure to treatment with SSRIs [99], or, intriguingly, by re-exposure to nicotine; suggesting that the repression of these negative emotional states may also be a driver for continued nicotine use in adulthood for those that started as adolescents. How exposure to nicotine in adolescence leads to these enduring behavioral changes, which resemble a model of psychiatric vulnerability, remains under investigation – but may result from enduring nicotine-induced changes to developing cholinergic and dopaminergic circuits, and the interplay between them.

## Enduring effects of nicotine in adolescence on adult cholinergic signaling

Acetylcholine acts as a neuromodulator in the brain, where it signals through cationic nAChRs and metabotropic muscarinic receptors, notably regulating cellular excitability, synaptic plasticity, and the coordinated firing of groups of neurons to play a key role in fundamental aspects of brain physiology including emotion, cognition and motivation [100]. Impairments to cholinergic signaling in the adult human brain have been linked to psychiatric disorders in adult patients, including schizophrenia, anxiety, and depression [101–103]. Notably, anomalies in the availability or expression of specific nAChR subunits have been implicated in depression [104] and schizophrenia patients [105, 106]. Whether these alterations to the expression and function of nAChRs are antecedent to the onset of these associated disease states remains an open question. Mouse models have begun to address how manipulating signaling through nAChRs in adult animals may produce symptoms associated with psychiatric diseases other than addiction to nicotine itself [74, 107–110]. However, in addition to its functions in the mature brain, ACh signaling is essential for neuronal development, where it plays a trophic role in establishing and maintaining neuronal connectivity [111, 112]. Thus, developmental exposure to nicotine stands to significantly perturb neuronal maturation by interfering with ACh signaling. In this section, we review evidence from animal models that exposure to nicotine in adolescence produces enduring alterations to nAChR expression and function, altering basal cholinergic signaling, response to later nicotine exposure, and establishing a vulnerable psychiatric phenotype.

Exposure to nicotine in adolescence is associated with brain-wide changes to the expression of discrete nAChR subunits, with some transient changes appearing during treatment or withdrawal, but with some persistent changes enduring until adulthood. Transient changes in nAChR expression following adolescent nicotine exposure have been reviewed elsewhere [44, 53, 113, 114]. Persistent changes to cholinergic and nicotinic signaling through nAChRs induced by nicotine in adolescence are of particular interest in the context of long-term psychiatric vulnerability, however there is less data available concerning this topic. Two weeks of exposure to nicotine in mid-adolescence (PND 30–47) increased binding to α4β2-containing nAChRs, an effect that persisted into adulthood in the PFC and midbrain [56, 63, 115], while an upregulation of α7- containing NAChRs in the striatum, brainstem, and cerebellum was apparent only transiently [116]. A similar mid-adolescent exposure regimen (PND 34–43) lead to an increased expression of α5, α6, β2, and β3 nAChR subunits in the adult VTA –– while the same exposure, given instead during adulthood, only increased the β3 subunit [117]. Brief exposure to nicotine in early adolescence (PND 27–33) increased nAChR function seven weeks later in the striatum, PFC, hippocampus, and thalamus/midbrain, including the VTA [118]. Nicotine treatment in mid-adolescence (PND 35–44) had sex-specific long-term effects on nAChR protein levels in the PFC, with α7 and β2 protein reduced in adult males (>P75) exposed to nicotine in adolescence and no change the expression of these subunits in female rats exposed at the same age [98].

Choline acetyltransferase (ChAT), an enzyme essential for ACh synthesis, is often used as a measure for cholinergic innervation. Changes in ChAT activity following exposure to nicotine in adolescence appear to be primarily transient, with an upregulation of ChAT activity in the striatum of male rats and in the midbrain of females reported during and shortly following exposure in mid-adolescence (PND 30–47), which were no longer detectable once the rats reached adulthood [119]. Similarly, oral exposure to nicotine in C57BL/6 mice in mid-adolescence (PND 30–45) increased ChAT activity when measured shortly after treatment at PND 50, an effect which normalized by PND 75 [63]. Persistent changes have been reported in the midbrain, where ChAT activity was reduced during and after nicotine injections during mid-adolescence (PND 30–47), and this reduction persisted until at least PND75 [115, 120].

## Persistent effects of adolescent nicotine exposure on DA circuits

DA neurotransmission is essential for diverse behavioral outputs, including motivation, cognition, reward learning, decision making, salience attribution, and voluntary motor control [121–127]. With such varied outputs, it is unsurprising that DA signaling has been heavily implicated in psychiatric disease, and neuroimaging studies in adult patients with schizophrenia, depression, and addiction have noted alterations to dopaminergic receptor expression [128–131]. Functional studies in adult rodent models have linked genetic or experimentally induced alterations in DA neuron activity, DA release, and receptor binding to transdiagnostic changes in motivation and cognitive control [49, 132–135]. As the dopamine system undergoes a period of profound and dynamic development in adolescence [39], it is increasingly proposed to function as a ‘plasticity system’, where experiences can create enduring changes to behavior through their actions on the dopamine system [78, 136]. Nicotine in adolescence is thus poised to alter DA development, as it acts directly on VTA DA neurons through their expression of nAChRs, modulating their firing rate in a population-specific manner in adult rodents [72, 137–139]. Nicotine also has indirect or circuit effects on DA neurotransmission, as nAChRs are also present on DA axon terminals, on neurons present in dopaminoceptive regions, and importantly on VTA GABA neurons, which regulate DA neuron activity [52, 68, 69, 140]. Nicotine may therefore alter interact with the developing DA system at multiple levels to promote later psychiatric vulnerability.

Nicotine in adolescence has been proposed to evoke enduring structural and functional changes in DA neurons and/or in their terminal regions. At the level of the VTA, basal DA neuron firing was increased in adult, male rats following exposure to nicotine in mid-adolescence (PND 35–44) [96, 98], however another group reports that basal DA neuron firing rate was decreased in adult rats following mid-adolescent (PND 38–42) nicotine exposure [141]. These disparate findings may result from differences in rat strains as Jobson, Ng, and colleagues studied Sprague-Dawley rats, while Cadoni and colleagues noted a decrease in firing rate in Lewis rats, with no significant change in Fisher rats. Structural analysis of VTA neuron populations note that nicotine in adolescence increased the proportion of DA neurons that do not express the vesicular glutamate transporter (TH+Vglut- neurons) in the posterior VTA in rats [142]. In this case, rats were exposed to nicotine starting at PND28, and the exposure continued until they were examined as adults, raising the question of when exactly these changes may occur, particularly as no comparison was made with rats whose exposure began as adults. Finally, nicotine-induced changes to local GABA signaling in the VTA may influence DA neuron function, as the concerted action of nicotine on both DA and GABA neurons in the VTA is necessary for nicotine reward [140]. Accordingly, a recent study found that adolescent, but not adult, nicotine exposure alters GABA signaling in the VTA by reorganizing chloride homeostasis, and, in turn, increases inhibitory tone over lateral VTA DA neurons [143].

At the terminal level, regional differences in DA function in the NAc and PFC, the terminal regions of the mesocorticolimbic DA pathway, have been implicated in rodent models of mood disorders and addiction. In the NAc, DA D1 receptors (D1Rs) were downregulated in the shell subregion of adult male rats that were exposed to nicotine in mid-adolescence (PND 35–44), while, in contrast, D1Rs in the NAc shell were enduringly unregulated in female rats exposed to nicotine in adolescence [97, 98]. Downregulation of D1R expression in the NAc shell of adult male rats following adolescent nicotine exposure was further associated with functional changes to local NAc medium spiny neurons (MSNs). Nicotine in adolescence produced a persistent hyperactive firing state in MSNs, albeit with decreased bursting activity [97]. Nicotine in adolescence has also been shown to change dendrite morphology in DAceptive regions including the NAc [144].

DA terminals in the PFC are known to develop throughout adolescence, and their development shapes the maturation of local circuits and calibrates adult cognitive function [83, 145]. Exposure to nicotine in mid-adolescence (PND 34–43) has been shown to increase electrically evoked DA release in the PFC of adult animals [146], an effect that was associated with enduring cognitive deficits including reduced attention and increased impulsivity. This exposure pattern also enduringly altered PFC long term potentiation (LTP) into adulthood, marked by an increased ability of prefrontal synapses to undergo spike-timing-dependent LTP [147]. These electrophysiological and behavioral alterations were associated with transient changes in nAChR expression [57], and enduring changes in mGluR2 signaling [147, 148]. Nicotine exposure in mid-adolescence (PND 35–44) has been shown to have sex-dependent effects on PFC function, as it increased the firing frequency of PFC pyramidal neurons and decreased PFC D1R protein expression in only in male rats [96, 98]. In contrast, this treatment increased D2R expression in the PFC of male rats, while decreasing D2R expression in the PFC of female rats [97, 98]. Finally, exposure to nicotine in mid-adolescence (PND 30–47) increased DA turnover in the and PFC at PND 80 in males and female rats [149], highlighting that female rodents should not be considered invulnerable to the effects of nicotine in adolescence, even if the changes produced by exposure are not the same as those seen in their male counterparts.

Together, these nicotine-induced changes to the developing DA system are thought to potentiate the effects of re-exposure to nicotine in adulthood and lead to the expression of behaviors associated with psychiatric vulnerability, yet few studies have made a causal link between these ideas. Our recent study showed that exposure to nicotine in early adolescence (PND 21–28), but not the same nicotine exposure in adulthood (PND 60 + ), produced enduring alterations in sensitivity to nicotine, notably defined by an increased sensitivity to its rewarding effects and a decreased sensitivity to its anxiogenic effects. These behavioral outcomes were linked with an electrophysiological signature of hyper-reactivity to nicotine specifically in the VTA-NAc circuit, and not in the VTA-Amygdala circuit, despite the clear role of this pathway in the anxiogenic effects of nicotine [137]. We next provided causal evidence linking nicotine-induced hyperactivity in the VTA-NAc circuit with the reduced anxiogenic effect of nicotine seen in adult mice exposed to nicotine in adolescence, as chemogenetic dampening of this specific pathway unmasked an adult-like behavioral response to nicotine [150]. This is, to our knowledge, the first instance where intervention at the level of circuit changes resulting from adolescent nicotine exposure rescued the expected adult behavior.

## Does nicotine in adolescence “freeze” the brain in an immature state ?

**Fig. 3:**
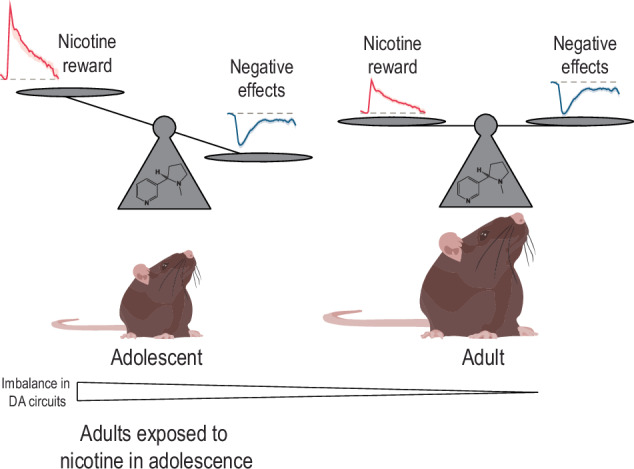
Nicotine exposure in adolescence leads to immature response to nicotine in adulthood. Adolescent mice show an increased rewarding effect of nicotine, paired with a blunted anxiogenic effect of the drug in comparison to adults. These differences result from an imbalance in dopamine neuron response to nicotine at the circuit level [150]. Adult mice that were exposed to nicotine in adolescence show similar behavioral and electrophysiological responses to nicotine, suggesting that adolescent exposure arrests the development of dopaminergic circuitry.

How exactly nicotine in adolescence alters DA circuit development is of primary interest. Exposure to stimulant drugs such as amphetamine, for example, has been shown to disrupt adolescent dopamine development, that is it produces outcomes not typically seen in control animals at any age. For example, amphetamine in adolescence misroutes a subset of dopamine axons to the PFC in an age- and sex-dependent manner, leading to a pathological ectopic innervation associated with cognitive deficits [51]. In contrast, nicotine exposure in adolescence appears to “freeze” dopamine circuitry in an adolescent-like state. This idea was first proposed as mice exposed to nicotine in adolescence showed behavioral responses to methylphenidate as adults that closely resembled the responses of naïve adolescent animals [151]. Recently, our work has given further mechanistic insight into this idea. We showed that exposure to nicotine in early adolescence, but not the same exposure in adulthood, produced a persistent vulnerability profile: adult mice that were exposed to nicotine in their adolescence consumed more nicotine, were less affected by the drugs negative effects, and experienced an increased sensitivity to its rewarding properties. What was interesting, however, is how closely this vulnerability profile mirrored the response of naïve adolescent mice to nicotine. Notably, adolescent mice were more sensitive to the rewarding effect of nicotine, showing a place preference for nicotine at a dose too low to produce this response in naïve adult mice. Adolescent mice were also impervious to the anxiogenic effect of nicotine, which seems to come online in parallel to the maturation of DA circuitry. Adult mice treated with nicotine in adolescence also showed immature electrophysiological responses to nicotine, with an adolescent-like exaggerated activation of VTA-NAc DA neurons. We thus hypothesized from these findings that exposure to nicotine in adolescence prolongs a naturally-occurring developmental imbalance in dopaminergic signaling between NAc- and amygdala-projecting pathways (Fig. 3). This imbalance, in turn, creates a vulnerable state by dampening negative effects of the drug (e.g. its anxiogenic effects). Finally, we were able to restore the mature behavioral response to nicotine in adolescent-exposed mice by chemogenetically resetting an adult-like balance in dopamine signaling in response to nicotine. We thus came to the initially unexpected conclusion that nicotine does not *disrupt* the adolescent development of dopamine pathways, per se, but rather arrests their development in an immature state [150].

## Limitations

Research examining the impact of nicotine during adolescence is critical for understanding how early exposure affects brain development and behavior. When holistically assessing the findings reported to date, however, it is apparent that there are significant limitations which must be taken into account when discussing the existing literature – equally in the case of conflicting findings and in the case of harmonious ones:

- *Adolescent ages*: As discussed earlier in the review, the definition of adolescence – and particularly of adolescence in animal models- does not necessarily have a strict consensus on its age range. We take care in this review to explicitly state the age ranges of each nicotine treatment, but it is important to keep in mind that the same treatment at different ages can certainly produce disparate outcomes. In the case of amphetamine exposure in adolescence, for example, exposure in early adolescence had opposite sex-dependent outcomes as exposure in mid-adolescence [51]. To the best of our knowledge, no study to date has yet directly compared the consequences of the same nicotine exposure at different adolescent ages. A final, important point on the issue of treatment ages is that not all studies include an adult control group, which is necessary to disentangle the developmental nature of long-term nicotine effects. Without this important control, it is impossible to conclude that the results of an adolescent exposure paradigm are specific to this exposure age.
- *Variability in nicotine exposure*: The routes of administration and doses of nicotine in studies can vary widely (Table 1, [152]. Different administration routes and/or nicotine doses can thus lead to significant variability in reported findings, particularly as different administration routes are known to alter the pharmacokinetic and pharmacodynamic properties of the same drug dose [153]. Indeed, this variability makes it difficult to directly compare results across studies or to replicate the natural patterns of nicotine use in human adolescents.

**Table 1:** Treatment details for manuscripts addressing persistent consequences of adolescent nicotine exposure.

Paper	doi	species	strain	sex	adolescent exposure age (PND)	adolescent exposure method	adolescent exposure dose
Abreu-Villaça et al. [115]	10.1038/sj.npp.1300221	rat	SD	M & F	30–38	minipump OR s.c.	0.6, 2, or 6 mg/kg/d
Adriani et al. [117]	10.1523/jneurosci.23-11-04712.2003	rat	SD	M	34–43	i.p.	0.4 mg/kg/d
Cadoni et al. [141]	10.1111/adb.12803	rat	Lewis and Fisher	M	38–42	s.c.	0.4 mg/kg (1× day for 5 days)
Counotte et al. [57]	10.1096/fj.11-198994	rat	wistar	M	34–43	s.c.	0.4 mg/kg (3× day)
Counotte et al. [146]	10.1038/npp.2008.96	rat	wistar	M	34–43	s.c.	0.4 mg/kg (3× day)
Counotte et al. [148]	10.1038/nn.2770	rat	wistar	M	34–43	s.c.	0.4 mg/kg (3× day)
Goriounova and Mansvelder, [147]	10.1523/jneurosci.5502-11.2012	rat	wistar	M	34–43	s.c.	0.4 mg/kg 3× daily × 10 days
Hudson et al. [97]	10.1111/adb.12891	rat	SD	M	35–44	s.c.	0.4 mg/kg 3x daily × 10 days
Iñiguez et al. [99]	10.1038/npp.2008.220	rat	SD	M	30–44	s.c.	(0.16, 0.32, and 0.64 mg/kg) twice daily
Jobson et al. [96]	10.1093/cercor/bhy179/5074516	rat	SD	M	35–44	s.c.	0.4 mg/kg 3× daily × 10 days
Kota et al. [118]	10.1016/j.bcp.2009.06.099	mice	ICR	M	27–33	s.c.	0.5 mg/kg per inj 2× day/14 days
Ng et al. [98]	10.1038/s41386-024-01853-y	rat	SD	M & F	35–44	s.c.	0.4 mg/kg 3× daily × 10 days
Nolley and Kelley [151]	10.1016/j.ntt.2006.09.026	mice	C57BL/6 J	M	25–57	i.p.	0.3 mg/kg 3.0 mg/kg
Reynolds et al. [150]	10.1101/2023.10.28.564518	mice	C57BL/6 J	M	21–28	oral	100 ug/ml
Ribeiro-Carvalho et al. [63]	10.1016/j.neuroscience.2009.05.032	mice	C57BL/6	M & F	30–45	oral	50 ug/ml in 2% sac
Slotkin et al. [119]	10.1016/j.brainresbull.2007.12.009	rat	SD	M & F	30–47	minipump	6 mg/kg/d
Slotkin et al. [116]	10.1016/j.brainres.2004.10.009	rat	SD	M & F	30–47	minipump	6 mg/kg/d
Thomas et al. [143]	10.1016/j.celrep.2018.03.030	rat	LE	M	28–42	i.p.	0.4 mg/kg
Trauth et al. [56]	10.1016/s0006-8993(99)01994-0	rat	SD	M & F	30–47	minipump	6 mg/kg/d
Trauth et al. [149]	10.1016/s0006-8993(00)03227-3	rat	SD	M & F	30–47	minipump	6 mg/kg/d
Vrettou et al. [142]	10.1016/j.dadr.2023.100180	rat	wistar	M	28–63	s.c.	0.35 mg/kg 3×/wk 6 weeks
- *Sex differences*: Sex and gender differences in nicotine use have been reported in human populations [154–156], notably among adolescent users, as well as in rodent studies [157]. While some studies include rodents of both sexes, others may not adequately account for sex differences or may use only one sex, limiting the generalizability of the findings. These differences can be significant in understanding the broader impact on human adolescents.

## Open questions

This review underscores how even brief nicotine exposure during adolescence can lead to significant and lasting changes in the cholinergic and dopaminergic systems. However, it also highlights how much we have yet to discover about the underlying mechanisms. Further studies are needed, particularly those aimed at determining when in adolescence are the critical moments for nicotine exposure to engender its enduring negative effects. Another major question is indeed how the enduring effects of nicotine in adolescence are perpetuated in the adult brain. One possibility is changes to nAChR receptor trafficking and surface expression patterns in response to nicotine, which may be short- or long-lasting in response to nAChR desensitization or inactivation following exposure [158]. Exposure to nicotine in adolescence may also produce enduring epigenetic changes, like those seen after pre-natal and juvenile exposure to nicotine [159]. Here, we focused on persistent alterations in neuromodulatory circuit function and we end our review on a current, intriguing hypothesis: that nicotine in adolescence may arrest the development of DA systems. Whether this may be true also for ACh circuits or other neurotransmitter systems is currently unknown. Naturally occurring developmental differences between the adolescent and adult brain have been proposed to render adolescents more sensitive to drugs of abuse – in particular, by augmenting rewarding effects of drugs and/or diminishing their negative effects. Adolescents are also thought to be more sensitive to stress. These conjectures again raise the idea that prolonging an adolescent-like state may indeed increase psychiatric vulnerability in adults.

While ACh and DA systems clearly both undergo significant maturation in adolescence, thus making them targets of developmental nicotine exposure, a comprehensive understanding of how the maturation of one neuromodulatory system affects the other during this critical period warrants investigation. Both muscarinic and nicotinic ACh receptors are present on VTA dopamine neurons, as well as on cholinergic and glutamatergic terminals regulating different aspects of dopamine release in output sites, such as the striatum and NAc [160–162]. Conversely, D2 dopamine receptors modulate the activity of striatal cholinergic interneurons [75]. Although these systems mature at different rates, their intertwined regulation suggests that disruptions in one could influence the development and function of the other. There remains a need for longitudinal research that tracks the concurrent development of both systems. Such studies could reveal how early changes in one system might predict alterations in the other and their subsequent behavioral implications, offering valuable insights for psychiatric research.

## Conclusion

**Fig. 4:**
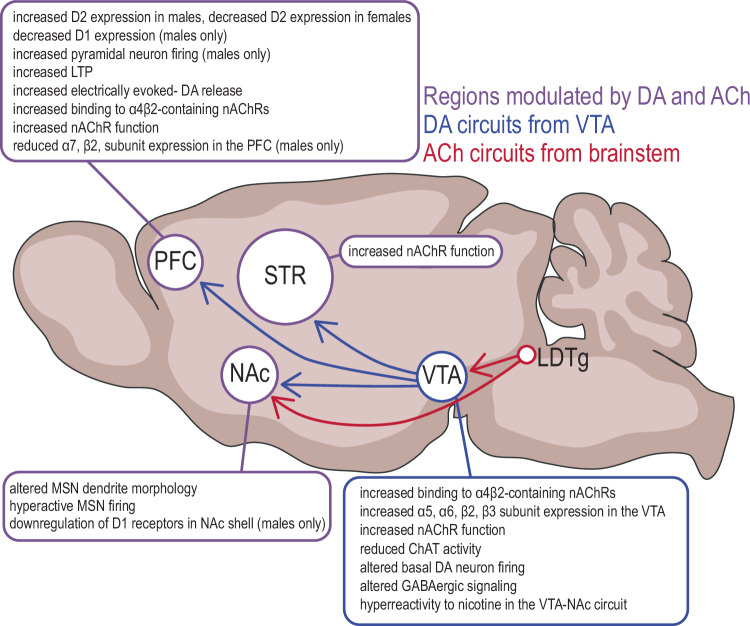
Summary of modifications in adult acetylcholine and dopaminergic systems induced by adolescent nicotine exposure. Alterations in functional outputs in adulthood, such as receptor expression and function, have been noted for both ACh and DA circuits following nicotine in adolescence. Sex-specific changes are noted where that information is available.

Nicotine use, and particularly nicotine use during adolescence is associated with an increased risk of addiction and mood disorders in adulthood. While prevention efforts over the past two decades have had some success at curbing adolescent initiation of cigarette smoking, recently vaping has emerged as an alternative nicotine delivery method immensely popular amongst adolescents [163]. While e-cigarettes are viewed as a safer alternative than conventional cigarettes, as they spare exposure to toxic chemical constituents of tobacco smoke, vapers are still chronically exposed to nicotine [164, 165]. Moreover, more adolescents are inclined to transition from vaping to conventional cigarettes, reversing a two-decade-long decline [166–169]. Thus, understanding the neurobiological underpinnings of how nicotine exposure in adolescence increases later psychiatric risk is essential for developing effective prevention and intervention programs to promote healthy transitions from adolescence to adulthood. In this review we cover the state of the literature with regards to studies of the long-term effects of nicotine in adolescence on neuromodulatory systems in animal models (summarized in Fig. 4), identify limitations in our ability to interpret these studies as a whole, and propose open questions that may drive the field forward.
